# Repetitive transcranial magnetic stimulation elevates the serum levels of neurotrophic factors and serotonin and its metabolites in patients with ischemic stroke

**DOI:** 10.3389/fneur.2025.1513131

**Published:** 2025-03-05

**Authors:** Wei Li, Wenyan Li, Yinghua Wen, Junying Wu

**Affiliations:** ^1^Department of Clinical Medicine, Shanxi Medical University, Taiyuan, China; ^2^Department of Rehabilitation Medicine, The First Hospital of Shanxi Medical University, Taiyuan, China

**Keywords:** rTMS, ischemic stroke, BDNF, cognitive function, depression

## Abstract

**Objective:**

Repetitive transcranial magnetic stimulation (rTMS) can effectively treat cognitive impairment in stroke patients; however, its mechanism of action remains unclear. The aim of this study was to investigate whether rTMS improves cognitive function by regulating the levels of brain-derived neurotrophic factor (BDNF), nerve growth factor (NGF), 5-hydroxytryptamine (5-HT), and 5-hydroxyindoleacetic acid (5-HIAA).

**Methods:**

In a 4-week study, 70 patients with ischemic stroke were randomly assigned to two groups: one received rTMS (*n* = 35) and the other received sham-stimulation (*n* = 35) in addition to conventional medication and rehabilitation training. Patients in the rTMS group were treated with rTMS at 10 Hz for 20 min per session. The Montreal Cognitive Assessment (MoCA) and response time in the n-back task were used to assess the severity of the disease. Fasting venous blood was collected in the early morning, both before and after the treatment. The peripheral blood levels of BDNF, NGF, 5-HT, and 5-HIAA were measured using the enzyme-linked immunosorbent assay (ELISA).

**Results:**

The levels of BDNF and NGF were higher in the rTMS group than in the sham group (*p* = 0.017, *p* = 0.008), after the rTMS treatment, and the levels of 5-HT and 5-HIAA were also elevated in the rTMS group (*p* = 0.049, *p* = 0.004). The changes in serum 5-HT and 5-HIAA levels after the rTMS treatment correlated with the changes in the MoCA and response time in the n-back task. There was a positive correlation between the serum 5-HT and BDNF levels (*r* = 0.4034).

**Conclusion:**

Our results showed that the BDNF, NGF, 5-HT, and 5-HIAA levels were upregulated after the rTMS treatment, which likely contributed to improvements in cognitive function and quality of life in the patients with stroke.

**Clinical trial registration:**

https://www.chictr.org.cn/showproj.html?proj=216761, ChiCTR2400082383.

## Introduction

Stroke is the second leading cause of disability and death worldwide, imposing a heavy burden on individuals, families, and society (1). Post-stroke cognitive impairment (PSCI) includes deficits specific to the site of the stroke lesion and deficits that may have occurred prior to the stroke (2, 3). Meanwhile, deficits in visuospatial, attentional, mnemonic, and executive functions are more closely associated with classic vascular cognitive impairment (VCI) and are often detected during post-stroke cognitive screening. It has been increasingly demonstrated that repetitive transcranial magnetic stimulation (rTMS) can modulate the neural excitability of brain regions (4) and has superior effects on cognitive functions compared to other non-invasive stimulation techniques. However, the mechanisms underlying rTMS-mediated cognitive function improvement remain largely unclear that may be inextricably linked to blood factors.

In addition to playing an important role in Alzheimer’s disease and memory impairment in patients with traumatic brain injury (TBI), brain-derived neurotrophic factor (BDNF) and nerve growth factor (NGF) are extremely important for the rehabilitation of patients with stroke (5, 6). BDNF binds to its high-affinity receptor, tropomyosin receptor kinase B (TrkB), to exert a significant physiological role in the brain. It is broadly and strongly expressed in the cortical and hippocampal regions, which are critical for learning and memory (7). In contrast, NGF is the most typical neurotrophic factor (NTF) that regulates neuronal survival, differentiation, growth, and apoptosis by binding to both tropomyosin receptor kinase (TrK) and p75 neurotrophin receptor (p75NTR) (8). Therefore, the regulatory roles of BDNF and NGF in cognitive function (e.g., memory and learning ability) have been widely recognized and investigated. 5-hydroxytryptamine (5-HT, serotonin), a neurotransmitter, regulates many important physiological processes, such as body temperature, sleep, appetite, pain, and muscle movement. It is also involved in the regulation of brain functions, such as cognition and emotion (9). The widespread impairment of serotonin and its metabolite, 5-hydroxyindoleacetic acid (5-HIAA), in patients with cognitive deficits suggests that serotonin disorders may further exacerbate delirium and cognitive impairments (10). Previous studies have shown a positive correlation between 5-HIAA and 5-HT levels as 5-HIAA is a urinary metabolite of 5-HT that has been widely shown to reflect changes in the 5-HTergic system (11). There is increasing evidence that serum levels of BDNF, NGF, serotonin, and its metabolite 5-HIAA correlate with rTMS. For example, BDNF (platelet-derived growth factor D), NGF, PDGF, and vascular endothelial growth factor (VEGF) levels were significantly higher in the rTMS group than in the untreated group 4 weeks after treatment (12). Among the studies on serotonin and its metabolites, chronic rTMS was found to upregulate presynaptic 5-HT in the rat brain (13) and increase hippocampal concentrations of 5-HT and 5-HIAA in Wistar rats following TMS (14).

Peripheral blood neurotrophic factors and serotonin and its metabolites are produced in the brain. After being secreted through the blood–brain barrier (BBB), they are released into the bloodstream by platelets (15). It has been reported that the passage of BDNF across the BBB occurs via a high-capacity, saturable transport system (16–18). Although 5-HT has difficulty crossing the BBB (19), the therapeutic mechanism of rTMS may be closely related to monoamine neurotransmitters, amino acid transmitters, and cortical loop systems. By modulating the excitability of the cerebral cortex, it increases the release of dopamine and 5-HT from striatal synaptosomes. The objective of this study was to investigate whether the serum levels of neurotrophic factors and serotonin and its metabolites are altered after rTMS treatment in patients with ischemic stroke and to assess whether there is a correlation between these factors and cognitive dysfunction. Based on previous studies (13, 14, 20), we hypothesized that serum levels of BDNF, NGF, 5-HT, and 5-HIAA are elevated after rTMS treatment and that changes in serum 5-HT and 5-HIAA levels are positively correlated with improvements in cognitive function. We also hypothesized that there is a correlation between serum 5-HT and BDNF levels after rTMS therapy. However, these topics have not been fully addressed in previous studies.

## Materials and methods

### Participants

Patients with ischemic stroke who were admitted to the Department of Rehabilitation Medicine at the First Hospital of Shanxi Medical University were recruited for this study between April 2024 and August 2024. They were included based on a diagnosis of stroke according to the diagnostic criteria established by the Fourth National Academic Conference on Cerebrovascular Disease (21) and were at their first stroke onset, confirmed using cranial CT or MRI. The duration of the neurological deficits exceeded 24 h, and their depression and cognitive function were assessed using the Montreal Cognitive Assessment (MoCA) (22) and response time in the n-back task (23). Demographic and clinical data of the patients were retrieved from the hospital databases. Patients were excluded if they had metal or electronic devices implanted in their body, as these are contraindications to rTMS treatment, as well as unstable vital signs or an inability to cooperate in completing the assessment of disorders of consciousness and post-stroke aphasia. Patients with epileptic disorders, severe cardiac, pulmonary, hepatic, renal, or psychiatric conditions, or malignant neoplasms were also excluded. Patients with other organic or psychiatric diseases that could affect the therapy, including brain tumors, multiple sclerosis, Parkinson’s disease, Alzheimer’s disease, serious somatic illnesses that affect the heart, liver, and lungs, and posterior circulatory dysfunction, and those who were using psychotropic drugs, such as SSRI, SNRI, and tricyclic antidepressants, were also excluded. Written informed consent was obtained from all participants. This study was conducted in accordance with the guidelines of the World Medical Association (the Declaration of Helsinki) and its Code of Ethics.

The sample size was calculated using GPower 3.1.9.7 with a larger effect value of d = 0.77, a degree of certainty of 1-*β* = 0.8, and a test level of *α* = 0.05. According to the pre-set parameters, the calculations showed that a minimum of 28 participants were required. Considering a potential failure rate of approximately 20%, 35 patients were included in each group.

### Randomization, blinding, and treatments

A randomized, blinded, parallel, sham-controlled design was employed to compare active versus sham rTMS interventions. A computer-generated randomization scheme was used to randomly assign the patients to the rTMS and sham groups (*n* = 35 each). A research assistant (independent of recruitment) concealed the allocation sequence using sealed, opaque envelopes. The investigators, clinical/cognitive raters, and participants remained blinded to the treatment condition. Before the rTMS therapy, the patients in both groups were administered conventional medications (including antiplatelet agents, lipid regulation and plaque stabilization medications, blood pressure-lowering drugs, blood glucose-lowering drugs, and other medications, such as aspirin, clopidogrel, amlodipine, indapamide, metformin, and acarbose) and regular rehabilitation training (including physiotherapy, occupational therapy, and cognitive training) for 28 days. rTMS was performed using a YRD CCY-1 type figure-of-eight coil connected to a transcranial magnetic stimulator, manufactured by Yiruide Medical Devices, Wuhan, according to the manufacturer’s safety and operation protocols, and administered by a skilled operator. rTMS was performed at 10 Hz with a 4 s interval for 20 sequences of stimulation. Each session lasted 20 min, once a day, seven days a week, for 4 weeks, at 80% of the resting motor threshold (RMT), which was determined by a physician not involved in the current study. The coils were securely fastened to the patient’s scalp at a 45-degree angle, targeting the dorsolateral prefrontal cortex (DLPFC). The patients in the sham rTMS group were placed in a single-wing tilt position using the same parameters and site to produce tactile and auditory stimulation with minimal direct brain effects (24).

### Clinical and cognitive assessments

Before and at the end of the 4-week treatment, the MoCA and response time in the n-back task were assessed, as previously described (22, 25). The MoCA is a commonly used clinical assessment tool for the rapid screening of mild cognitive dysfunction. It evaluates the following seven domains: visuospatial and executive functioning (5 points), naming (3 points), attention (2 points), language (3 points), abstraction (2 points), delayed recall (5 points), and orientation (6 points). The total score is 30, with scores of 26 and above indicating normal cognitive functioning.

E-Prime (version 2.0; Psychology Software Tools, Sharpsburg, PA, United States) was used to administer an auditory n-back working memory task. The patient was seated in front of a 13.3″ laptop monitor at a viewing distance of 55 cm in a well-lit room. In the n-back task, numbers 0–9 were randomly displayed at the center of a white screen, and the patient was asked to make a quick and accurate judgment by comparing the number displayed on the screen to the numbers preceding it as soon as the number appeared. In each task, 42 numbers (50% of the target) appeared sequentially in a fixed pre-randomized order, with each number presented at a fixed interval of 2,000 ms and 100 ms between numbers (25).

### Biochemical assessment

Fasting venous blood was collected from the patients at 7 am, both before and after the treatment. After centrifugation at 2000 rpm for 20 min, 100 μL of the serum was transferred to the wells of the microtiter plates and mixed with a biotin-coupled antibody solution. The levels of BDNF (cat. no. EH0043), NGF (cat. no. EH0242), 5-HT (cat. no. EH4005), and 5-HIAA (cat. no. EU2582) were measured using the enzyme-linked immunosorbent assay (ELISA) with the Human ELISA Kit (cat. no. FN231031) from Fenn biotech, Wuhan, China, according to the manufacturer’s instructions. The absorbance at a wavelength of 450 nm was read using a Multiskan™ FC microplate photometer (Thermo Fisher, United States).

### Statistical analysis

The data were processed using SPSS (version 27.0). The measurement data were expressed as mean ± standard deviation (SD) and compared using the independent samples *t*-test. For the enumeration data, the *χ* 2 test was used. Pairwise samples *t*-tests were used to compare intragroup and intergroup means. The correlations between the variables were analyzed using the Pearson correlation coefficient. A *p*-value of <0.05 was considered to be statistically significant.

## Results

### Patient characteristics

**Figure 1:**
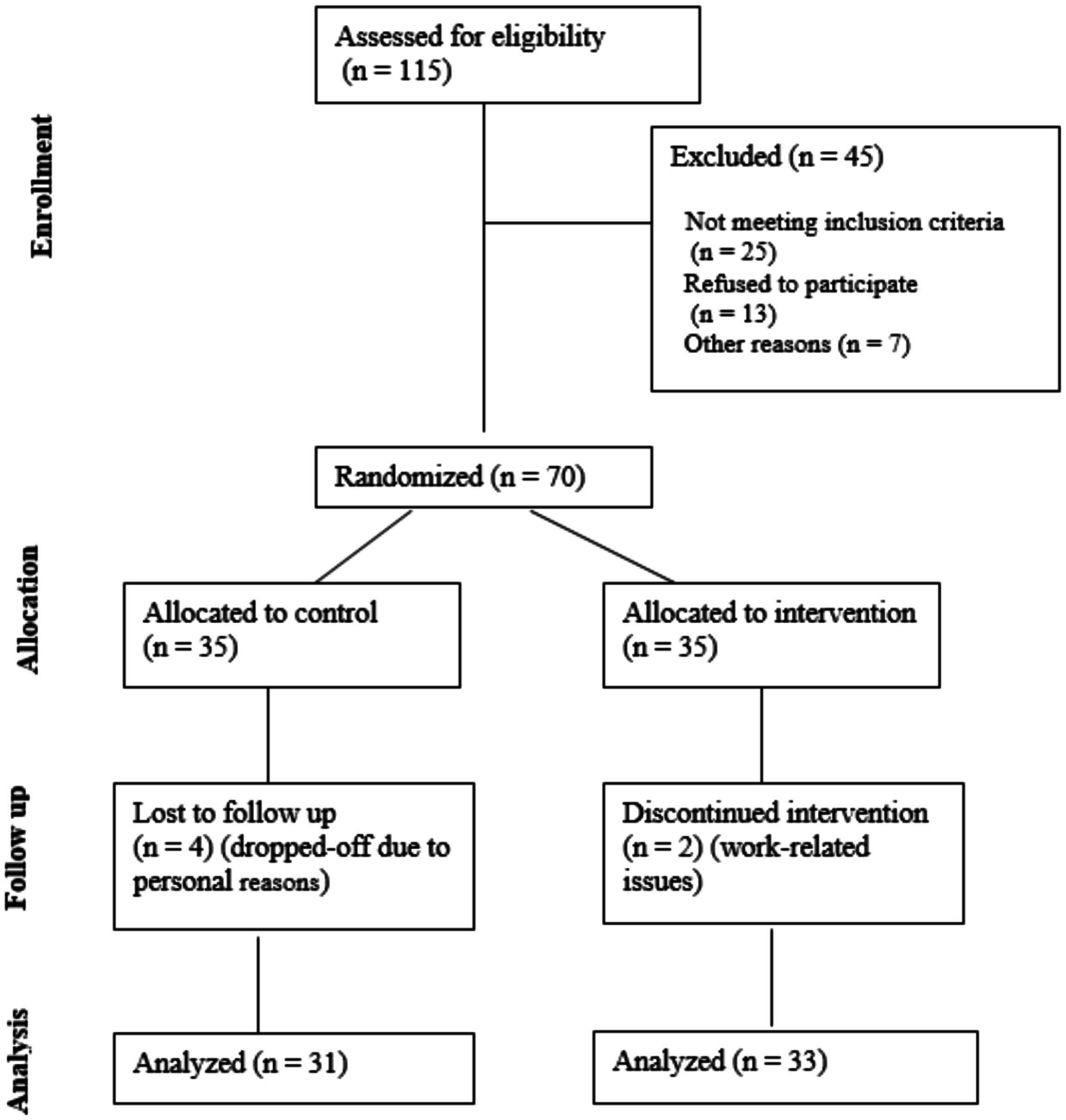
Consort flow diagram of participant recruitment and retention.

A total of 115 patients with ischemic stroke were initially identified, of whom 70 were randomized for intervention and control after excluding 45 due to failure to meet the inclusion criteria (25), refusal to participate (13), and other reasons (7). Four patients were dropped out after the assessment, and two patients did not complete the study. During the study, one patient was removed due to unstable vital signs starting on day 5, and blood samples were not obtained from three patients. Finally, data were analyzed for 33 patients in the rTMS group and 31 patients in the sham group (Figure 1). There was no statistically significant difference in age, sex, education level, and disease duration between the two groups (Table 1, *p* > 0.05).

**Table 1:** Comparison of the demographic and clinical characteristics between the rTMS and sham groups.

	rTMS (*n* = 35)	Sham (*n* = 35)	t/χ2	*p*
Age (years)	62.97 ± 9.36	62.53 ± 7.41	0.199	0.843^a^
Gender (male/female)	15/20	14/21	0.059	0.808^b^
Education (years)	8.57 ± 3.25	8.77 ± 2.53	−0.266	0.791^a^
Duration of disease (days)	13.80 ± 3.61	13.93 ± 3.72	−0.141	0.888^a^

a By independent samples *t*-test.

b By Chi-square test.

### Clinical response

**Figure 2:**
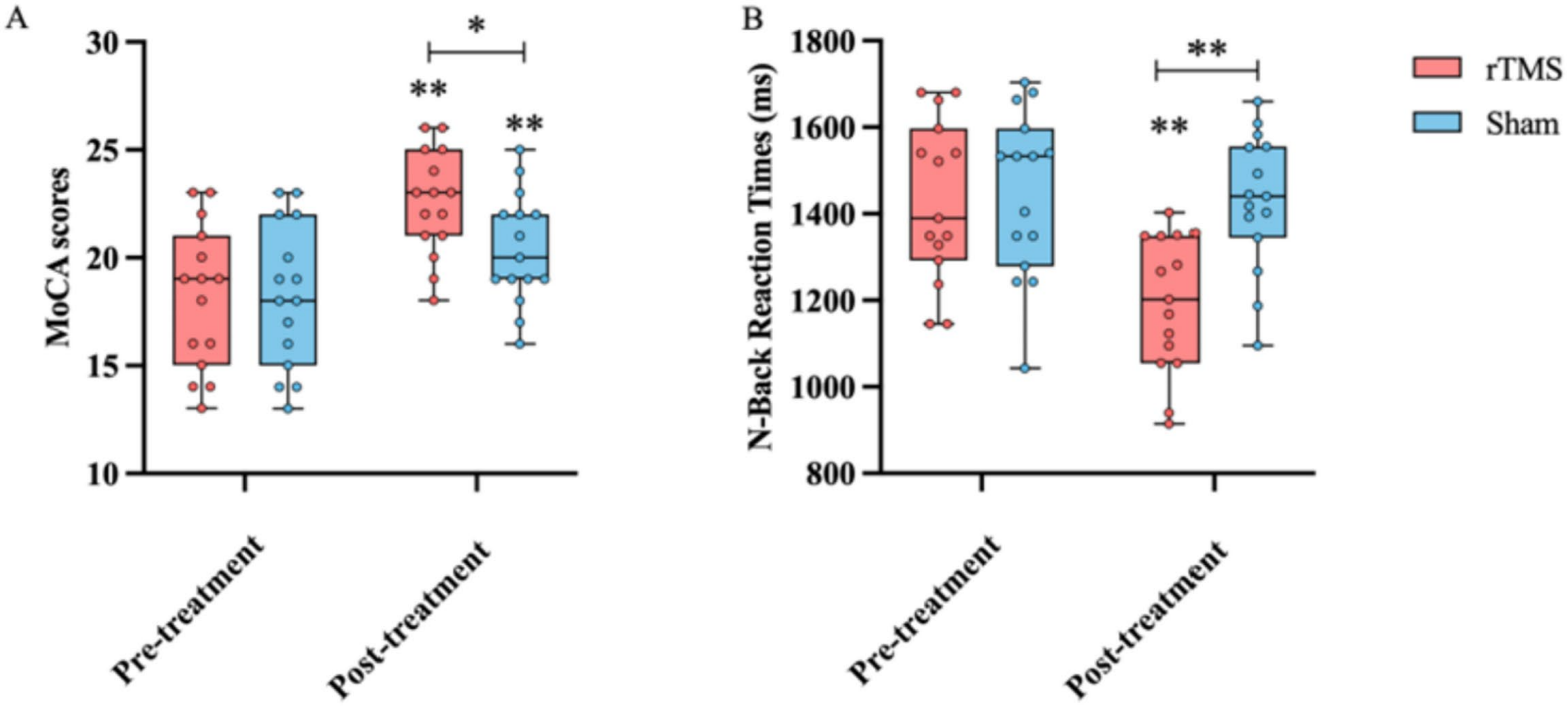
Comparison of the MoCA score **(A)** and response time in the n-back task **(B)** before and after the treatment, as well as between the rTMS and sham groups. * and ** indicate statistically significant intragroup or intergroup differences after the rTMS treatment (*p* < 0.05 and *p* < 0.001, respectively). BDNF, brain-derived neurotrophic factor; NGF, nerve growth factor; 5-HT, 5-hydroxytryptamine; 5-HIAA, 5-hydroxyindoleacetic acid; MoCA, Montreal Cognitive Assessment.

The assessments showed that at the end of the 4-week rTMS therapy, there was a significant increase in the MoCA score (24.53 ± 2.45 vs. 21.13 ± 3.34, *p* < 0.05) and a significant reduction in the response time in the n-back task (1323.67 ± 158 ms vs. 1589.69 ± 158, *p* < 0.01) in the rTMS group compared to the sham group. These parameters did not differ significantly between the rTMS and sham groups before the rTMS treatment (Figure 2), demonstrating that the rTMS treatment significantly improved cognitive ability.

### Serum BDNF, NGF, 5-HT, and 5-HIAA levels

**Figure 3:**
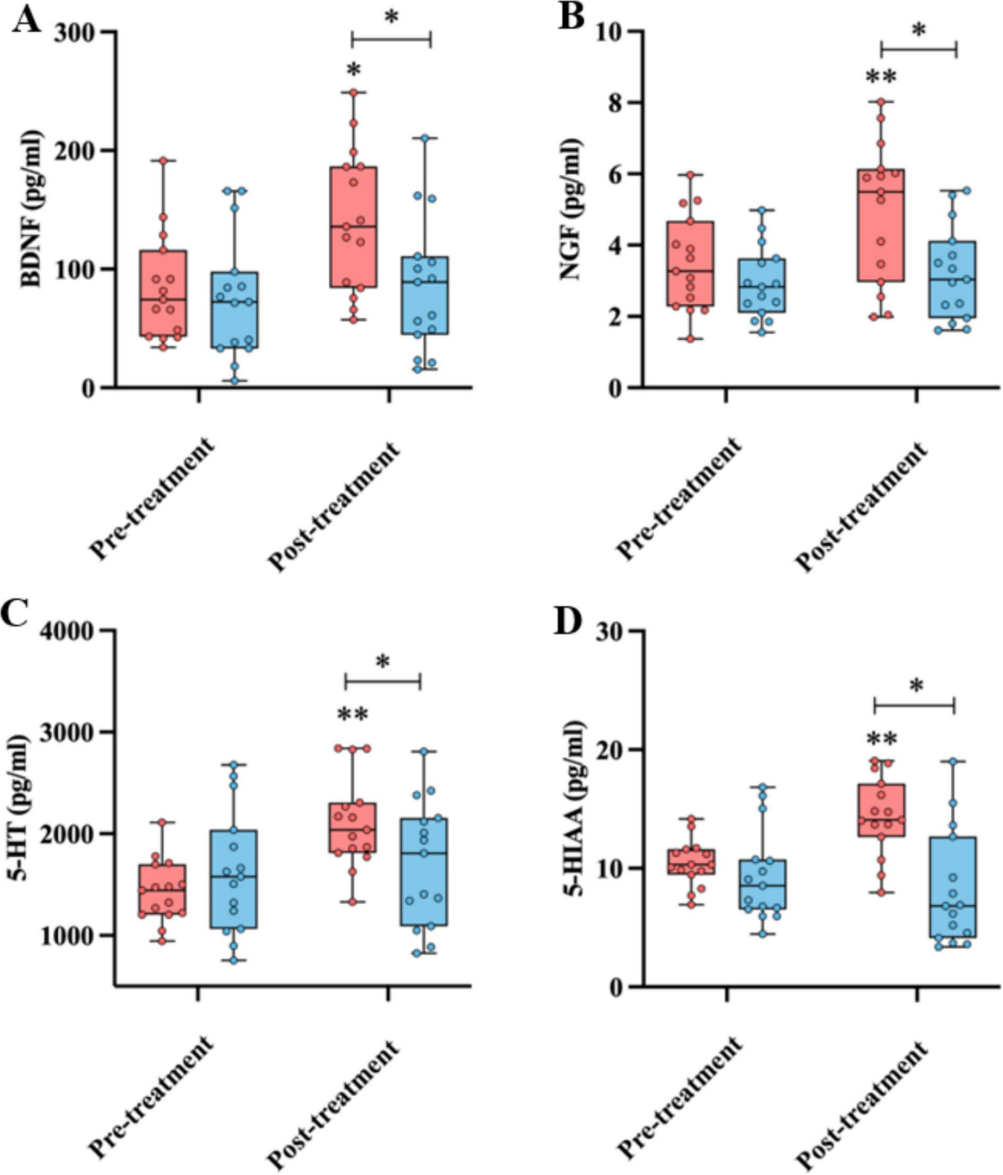
Comparison of the BDNF **(A)**, NGF **(B)**, 5-HT **(C)**, and 5-HIAA **(D)** levels before and after the treatment, as well as between the rTMS and sham groups. * and ** indicate statistically significant intragroup or intergroup differences after the rTMS treatment (*p* < 0.05 and *p* < 0.001, respectively). BDNF, brain-derived neurotrophic factor; NGF, nerve growth factor; 5-HT, 5-hydroxytryptamine; 5-HIAA, 5-hydroxyindoleacetic acid; MoCA, Montreal Cognitive Assessment.

The BDNF, NGF, 5-HT, and 5-HIAA levels were assessed before and after the rTMS treatment. The results showed that after 14 days of the rTMS treatment, the levels of BDNF (161.01 ± 59.96 vs. 116.77 ± 57.07 pg./mL, *p* < 0.05), NGF (6.95 ± 1.97 vs. 4.61 ± 1.32 pg./mL, *p* < 0.01), 5-HT (2212.62 ± 450.80 vs. 1707.28 ± 615.53 pg./mL, *p* < 0.05), and 5-HIAA (17.36 ± 3.31vs. 11.59 ± 4.87 pg./mL, *p* < 0.01) in the rTMS group were significantly higher than those in the sham group (Figure 3). However, the serum levels of these compounds in the sham group were not significantly different before and after the rTMS therapy (Figure 3).

### Correlation between blood 5-HT, 5-HIAA, MoCA, and response time in the n-back task

**Figure 4:**
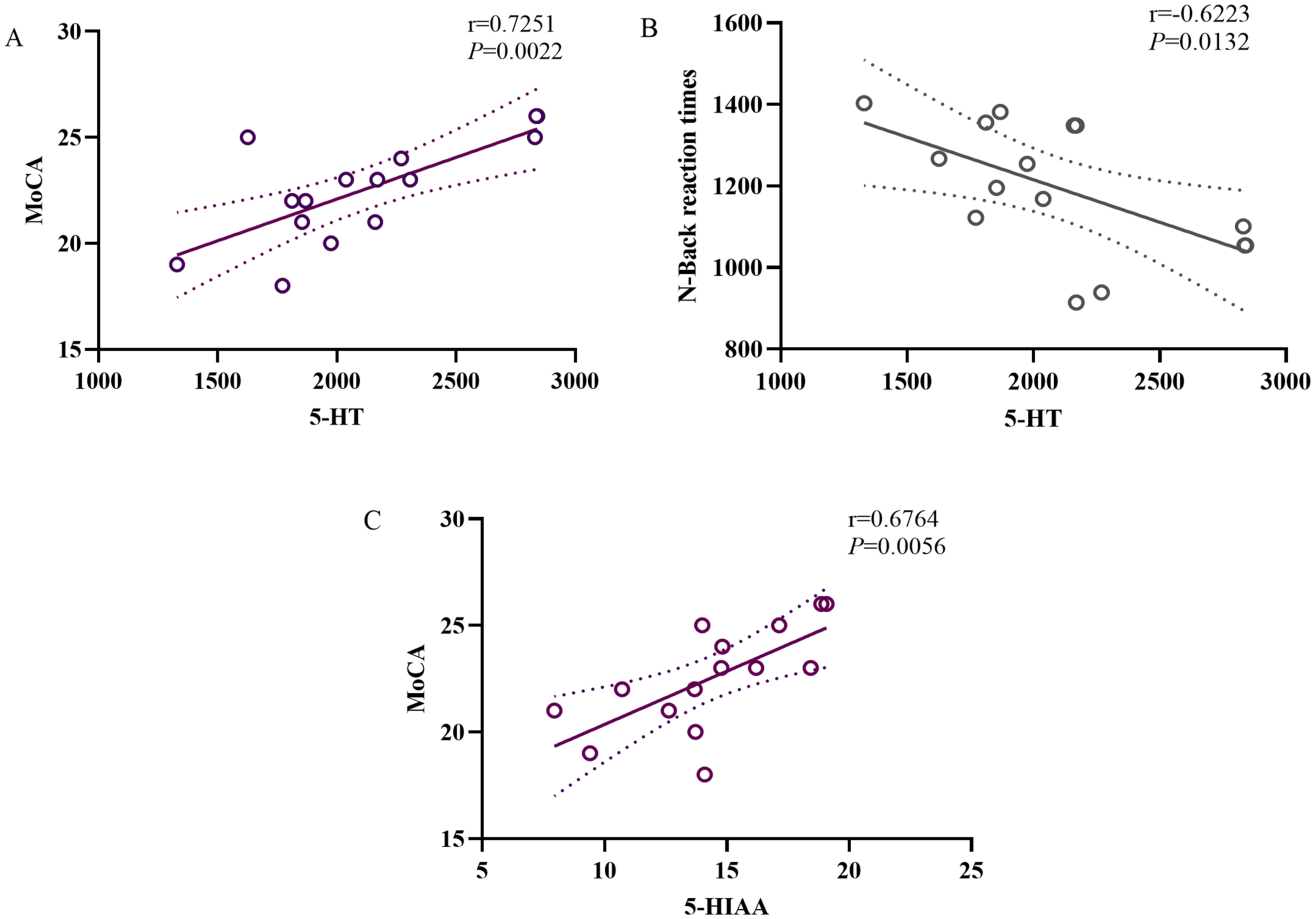
Correlations between blood 5-HT **(A)**, 5-HIAA **(C)**, MoCA scores, and response time in the n-back task **(B)** after rTMS. BDNF, brain-derived neurotrophic factor; NGF, nerve growth factor; 5-HT, 5-hydroxytryptamine; 5-HIAA, 5-hydroxyindoleacetic acid; MoCA, Montreal Cognitive Assessment.

There were correlations between the serum levels of 5-HT, MoCA scores, and response time in the n-back task in the rTMS group after the rTMS treatment (Figures 4A,B, *r* = 0.5258, *p* = 0.0022; *r* = 0.3873, *p* = 0.0132). The 5-HIAA levels were also correlated with the MoCA score (*r* = 0.6764, *p* = 0.0056) but not with the response time in the n-back task (Figure 4C). These effects were specifically observed in the rTMS group.

### Correlation between 5-HT and BDNF

**Figure 5:**
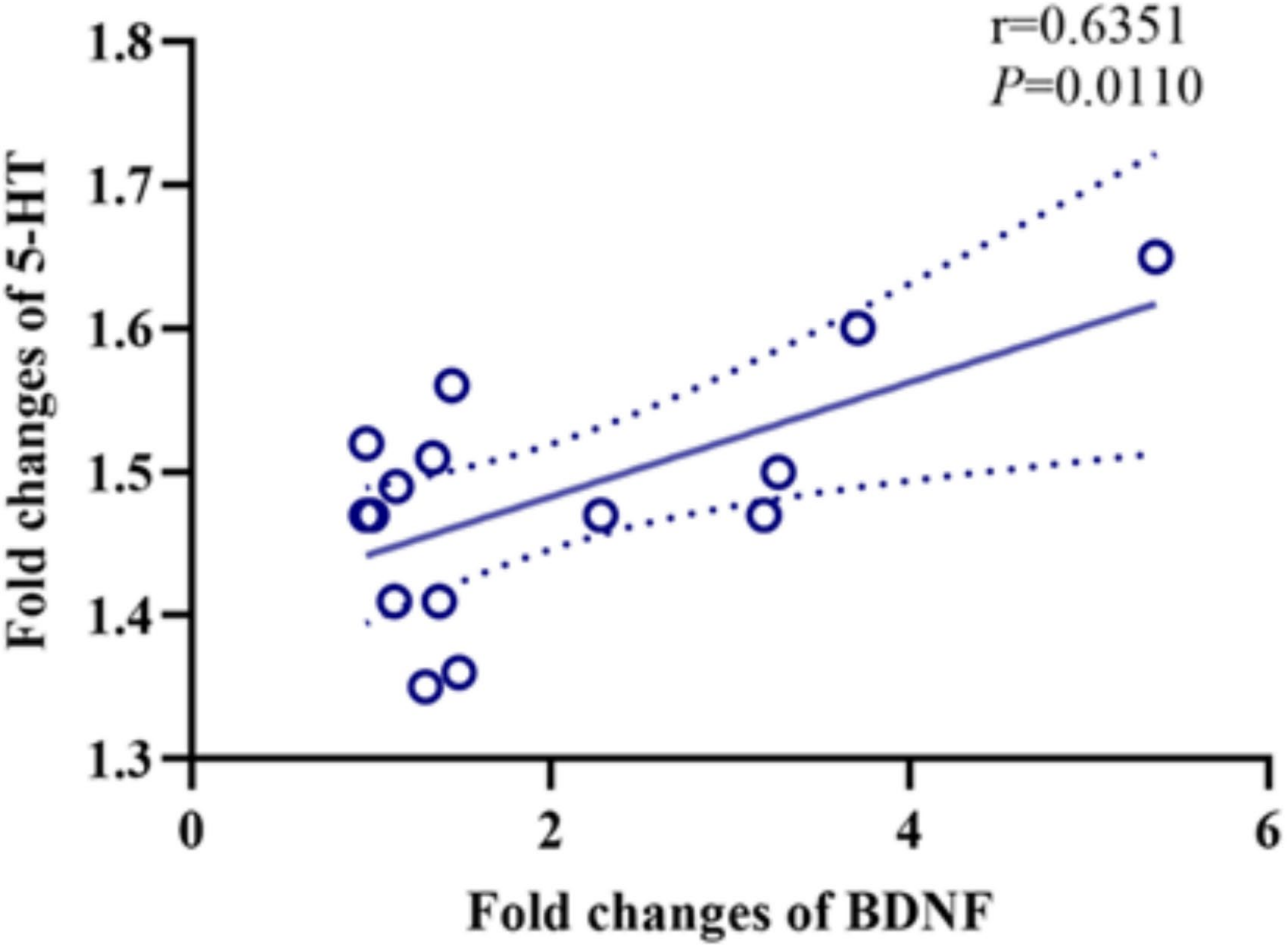
Correlation analysis of peripheral blood 5-HT with BDNF after rTMS. HT, 5-hydroxytryptamine; BDNF, brain-derived neurotrophic factor.

Both 5-HT and BDNF levels were increased in the rTMS group after the treatment. The correlation analysis showed a relationship between the fold changes in 5-HT and BDNF in the rTMS group before and after the treatment (Figure 5, *r* = 0.4034, *p* = 0.0110).

## Discussion

The purpose of the present study was to assess the effects of rTMS on the serum concentrations of BDNF, NGF, 5-HT, and 5-HIAA in patients with ischemic stroke. The results showed that the cognitive functions of the stroke patients improved after the rTMS treatment compared to the sham group. The serum levels of BDNF, NGF, 5-HT, and 5-HIAA were significantly higher after the rTMS treatment in the rTMS group than in the sham group. At the same time, the serum 5-HT and 5-HIAA levels were positively correlated with the MoCA score after the treatment, and the 5-HT levels were negatively correlated with the response time in the n-back task. The fold changes in the 5-HT levels after the treatment were positively correlated with those in the BDNF levels in the rTMS group. These results confirmed our current hypothesis that serum levels of BDNF, NGF, 5-HT, and 5-HIAA are elevated after rTMS treatment and are positively correlated with improvements in cognitive function in patients with stroke.

Luber et al. found that rTMS may promote cognitive recovery through the initiation of functionally recruited neurons, modulation of naturally oscillating brain behaviors, and induced neuroplasticity (26). This and previous studies have revealed that rTMS increases the serum concentrations of BDNF and NGF in patients (15, 27). TMS stimulates the glutamatergic expression of NGF and BDNF mRNAs in hippocampus neurons and activates glutamatergic circuits in the human motor cortex (28), promoting the repair and regeneration of damaged neurons. Findings from rTMS-treated rats have indicated that rTMS may preferentially enhance the expression of the BDNF–TrkB signaling pathway by augmenting the responsiveness of TrkB to BDNF, as well as by elevating the affinity between BDNF and TrkB (29). In addition, the increase in the serum BDNF and NGF levels observed in this study is consistent with results obtained in other studies examining cognitive function improvement through medical approaches, such as antidepressant drugs including selective serotonin reuptake inhibitors (SSRIs), electroconvulsive therapy (ECT), metal ions, and other substances (27, 30). This suggests that rTMS may improve cognitive function, depression, psychiatric disorders, and Alzheimer’s disease in patients by upregulating serum BDNF and NGF levels. On the other hand, plenty of evidence has shown that BDNF and NGF levels are reduced in patients with cognitive dysfunction (31, 32). The peripheral nervous system and central nervous system (CNS) in mammals contain BDNF, NGF, neurotrophic factor-3 (NT-3), and neurotrophic factor-4 (NT-4) (33). These factors are involved in neuronal plasticity and the maintenance of cell survival (33). Therefore, decreased BDNF and NGF levels may lead to atrophy of the hippocampus and prefrontal cortex, while upregulation of BDNF and NGF can promote neuronal regeneration, vascularization, and the expression of related proteins (34). The results of the present study suggest that the neurotrophic effect of rTMS can increase the expression of BDNF, thereby improving the therapeutic effect and enhancing cognitive function in rTMS-treated patients. However, contrary to our findings, no changes in BDNF and NGF levels were observed in other studies (35, 36). Further research is needed to determine the exact reasons for these differences.

rTMS increased the serum levels of 5-HT and 5-HIAA in the patients, which is in line with the findings of previous studies, as described in a meta-analysis of 65 clinical studies conducted from 1971 to 2022, which demonstrated that TMS increased serum 5-HT levels with moderate evidence (20). Several animal studies have shown that rTMS may affect the 5-HTergic system via the 5-HT1A receptor. For example, a single session of rapid rTMS on the rat frontal cortex resulted in a significant increase in the 5-HT1A receptor, as revealed using radiographic autoradiography at 24 h post-stimulation (37). Chronic rTMS decreased the ability of the 5-HT1A receptor agonist OH-DPAT to reduce the release of serotonin levels at the projection site (38). In clinical trials, HF- rTMS affected 5HT2A receptors in the DLPFC and hippocampus in different ways, which is consistent with the action of SSRIs (39). In patients with stroke, increased levels of 5-HT may also upregulate the levels of its metabolite, 5-HIAA, suggesting that rTMS can regulate 5-HIAA production by inducing 5-HT production. 5-HT regulates synaptic function in the adult brain, modulating the formation and growth of neural synapses and promoting cell survival. As an important compound in the neuronal signaling pathway, 5-HT not only improves memory and learning but also targets neurodegenerative diseases, such as Alzheimer’s disease and depression (40). SSRIs improve depressive symptoms by blocking the reuptake of 5-HT in neurons and increasing the concentration of 5-HT in synapses (41). Recently, it has been shown that 5-HT moves from its sources to neurons and astrocytes through energy gradients in the extracellular and cerebrospinal fluids, allowing 5-HT to diffuse and flow. This results in increased neuroplasticity and trophic activity upon binding to the receptor (42), which helps reduce anxiety and depression and improve cognitive functioning in patients (43). Thus, the increase in serum 5-HT levels stimulated by rTMS is likely associated with neuroplasticity. As a metabolite of 5-HT, the level of 5-HIAAmay be related to the levels and conversion of 5-HT (44). It has been well established that the levels of 5-HT and 5-HIAA are lower in patients with severe depression and cognitive impairment (45). However, it was also shown that the levels of 5-HIAA in the prefrontal cortex (PFC) and cerebrospinal fluid of rats treated with rTMS were decreased (46), which contradicts our findings. Further studies are needed to elucidate the possible reason for this discrepancy.

ROC curve analysis showed that the MoCA has a better discriminatory ability for cognitive deficits and a higher diagnostic value than the MMSE. In addition, the n-back task is used extensively in cognitive neuroscience (47). Therefore, we used these tools to examine cognitive function following rTMS. The results showed that the rTMS treatment had a significant effect on improving the cognitive function, emotional state, and memory attention of the patients (47) and that the level of 5-HT and 5-HIAA in the patients with cognitive, memory, and attention deficits after stroke were significantly lower (48). Patients with Parkinson’s disease and cognitive impairment were found to have lower MoCA and MMSE scores compared to patients with Parkinson’s disease without cognitive impairment (49). In contrast to earlier research, this study did not find a significant association between 5-HIAA and response time in the n-back task (50). In addition, we found that 5-HT might interact with BDNF to enhance the production and functionality of 5-HT in neurons. It has been demonstrated that 5-HT influences mood and cognition by regulating BDNF production and signaling (51). The activation of the 5-HT receptor cAMP-PKA pathway is coupled with the activation of transcription factors such as CREB and FKHR, which induce the transcription of BDNF genes (52). BDNF stimulates the growth and development of the axons of 5-HT neurons that dominate the encephalic cortex, potentially leading to an increase in the number of 5-HT synapses within this brain region (53). In addition, we found that the increase in the serum 5-HT concentration before and after the treatment was positively correlated with the BDNF concentration. Therefore, there may be an interaction between 5-HT and BDNF, leading to their synergistic regulation of neuronal plasticity and survival (54).

TMS combined with sertraline improves cognitive function, mood, learning ability, attention, and working memory in patients with depression. rTMS can promote cerebral cortical metabolism, increase cerebral blood perfusion, and regulate neural signaling, which, in turn, improves perceptual and memory abilities, because sertraline can regulate neurotransmitter expression and help enhance hippocampal nerve function (55). Although drug therapy remains the primary treatment for this disease, its effects are limited when used alone, and it needs to be combined with non-invasive neuromodulation technology for better patient recovery (56).

There are several limitations in this study. First, the sample size was small, and it was a single-center study, with the assessments performed at a single time point within a relatively short experimental period, without long-term follow-up. Second, due to the short treatment period at the hospital, the clinical response to rTMS therapy might not have been fully revealed, and a longer treatment period is needed to better understand the mechanism of rTMS on cognitive function. Third, all participants were taking different medications during the study period, which might have affected the results.

In conclusion, the present study demonstrated that rTMS was effective in improving the cognitive function and emotional state of the patients with ischemic stroke. In addition, the underlying mechanism may be closely related to the upregulation of BDNF, NGF, 5-HT, and 5-HIAA levels. rTMS may improve cognitive function and emotional state by promoting neural regeneration, enhancing the 5-HTergic system, and activating the brain-derived growth factor/tyrosine kinase receptor signaling pathway. Furthermore, BDNF and 5-HT may have synergistic effects on cognitive improvement in rTMS-treated patients.

## Data Availability

The original contributions presented in the study are included in the article/supplementary material, further inquiries can be directed to the corresponding author.
